# Population-based 10-year cumulative revision risks after hip and knee arthroplasty for osteoarthritis to inform patients in clinical practice: a competing risk analysis from the Dutch Arthroplasty Register

**DOI:** 10.1080/17453674.2021.1876998

**Published:** 2021-01-22

**Authors:** Maaike G J Gademan, Liza N Van Steenbergen, Suzanne C Cannegieter, Rob G H H Nelissen, Perla J Marang-Van De Mheen

**Affiliations:** a Department of Orthopaedics, Leiden University Medical Center, Leiden;; b Department of Clinical Epidemiology, Leiden University Medical Center, Leiden;; c Dutch Arthroplasty Register, ‘s Hertogenbosch;; d Department of Biomedical Data Sciences, Medical Decision Making, Leiden University Medical Center, Leiden, The Netherlands

## Abstract

Background and purpose — A lifetime perspective on revision risks is needed for optimal timing of arthroplasty in osteoarthritis (OA) patients, weighing the benefit of total hip arthroplasty/total knee arthroplasty (THA/TKA) against the risk of revision, after which outcomes are less favorable. Therefore, we provide population-based 10-year cumulative revision risks stratified by joint, sex, fixation type, and age.

Patients and methods — Data from the Dutch Arthroplasty Register (LROI) was used. Primary THAs and TKAs for OA between 2007 and 2018 were included, except metal-on-metal prostheses or hybrid/reversed hybrid fixation. Revision surgery was defined as any change of 1 or more prosthesis components. The 10-year cumulative revision risks were calculated stratified by joint, age, sex, at primary arthroplasty, and fixation type (cemented/uncemented), taking into account mortality as a competing risk. We estimated the percentage of potentially avoidable revisions assuming all OA patients aged < 75 received primary THA/TKA 5 years later while keeping age-specific 10-year revision risks constant.

Results — 214,638 primary THAs and 211,099 TKAs were included, of which 31% of THAs and 95% of TKAs were cemented. The 10-year cumulative revision risk varied between 1.6% and 13%, with higher risks in younger age categories. Delaying prosthesis placement by 5 years could potentially avoid 23 (3%) THA and 162 (17%) TKA revisions.

Interpretation — Cumulative 10- year revision risk varied considerably by age in both fixation groups, which may be communicated to patients and used to guide timing of surgery.

In Western countries about 10–23% of women and 6–15% of men receive a total knee arthroplasty (TKA) during their life. For total hip arthroplasty (THA) these numbers are 12–16% of women and 8–11% of men (Ackerman et al. 2017a, b). Although arthroplasty is an effective intervention, the optimal timing of arthroplasty is crucial given the long-term survival of the prosthesis is still limited.

To achieve optimal timing of primary THA/TKA, the benefit of surgery has to be weighed against the risk for revision surgery, which has less favorable outcomes (Petersen et al. 2015). Hence, when younger patients have lower revision risks than elderly patients, one might consider delaying surgery to optimize outcome from a lifetime perspective.

Valid prediction models providing individualized lifetime revision risks may help guide decision-making on optimal timing, but such models are rare (Prokopetz et al. 2012, Paxton et al. 2015). Valid revision risks can also be provided by simply calculating these risks in the population of interest. For example, Bayliss et al. (2017) modelled lifetime revision risks after TKA/THA with Clinical Practice Research Datalink data. However, they did not include implant (fixation) type or indication for surgery, while both may impact revision risks. Furthermore, most arthroplasty registries published cumulative revision risks, without taking the competing risk of dying into account. Also, stratification of revision risks into more than one/two subgroups was not performed, whereas this is important when providing personalized information.

We therefore provide 10-year cumulative revision risks for OA patients stratified by joint, sex, age, and fixation type, using data from the Dutch Arthroplasty Register and taking into account the competing risk of dying. We also estimate the number of potentially avoided arthroplasties by delaying TKA/THA by 5 years. Furthermore, we project our numbers onto the expected Dutch population in 2025/2035.

## Patients and methods

### Study design

This is a population-based cohort study.

### Data sources

The Dutch Arthroplasty Register (LROI) is a nationwide population-based registry that includes arthroplasties implanted in the Netherlands since 2007. The LROI was initiated by the Netherlands Orthopaedic Association (NOV), and has a completeness of reporting of over 95% for primary THA and TKA and over 88% of hip and knee revision arthroplasties up to 2013 (van Steenbergen et al. 2015), further increasing to 98% in 2017 (www.lroi-report.nl).

### Statistics Netherlands

Statline is the electronic databank of Statistics Netherlands, which publishes statistical information regarding various aspects of the Dutch population. Data from Statline (http://statline.cbs.nl) was used to project the number of revisions to the expected Dutch population by age and sex in 2025 and 2035. In this way we could quantify the impact on revision surgery of delaying primary arthroplasty by 5 years.

### Study population and definitions

All primary TKAs or THAs for OA from the LROI in the period 2007–2018 were included. Metal-on-metal THAs were excluded (n = 5,518, 2%), as these are not used anymore due to the high failure rates. Only prostheses with cemented or uncemented fixation were included and patients with missing data were excluded (Figure). For TKA and THA median length of follow-up was respectively 4.2 years (IQR 4.9) and 4.3 years (IQR 5.1), with a maximum of 12 years in both groups. Age at primary surgery, sex, fixation type (uncemented or cemented), and time between primary surgery and revision, death, or end of follow-up as well as status (revision, death, or alive without revision) were extracted from the LROI database. Revision surgery was defined as any change (insertion, replacement, and/or removal) of one or more components of the prosthesis.

**Figure:**
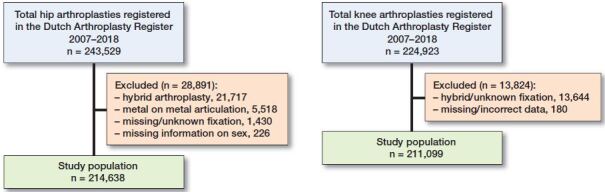
Flow chart of study population, total hip arthroplasty (left panel) and total knee arthroplasty (right panel).

### Statistics

Baseline characteristics stratified by joint were summarized using mean (SD) for continuous outcomes or number with percentage for categorical outcomes. All confidence intervals (CI) given represent the 95% confidence interval.

Survival time of the implant was calculated as the time from primary THA or TKA to first revision arthroplasty for any reason, death of the patient, or January 1, 2019. Cumulative revision risk within 10 years was calculated using competing risk analysis, where death was considered a competing risk (Lacny et al. 2015, Wongworawat et al. 2015). These cumulative revision risks were calculated stratified by joint, age at primary arthroplasty, sex, and cemented/uncemented fixation of the prosthesis. Cumulative risks within 10 years were not given if at 10 year less than 20 patients were at risk. Age at primary arthroplasty was categorized into to the following predetermined groups: 50–54, 55–59, 60–64, 65–69, 70–74, 75–79, 80–84 and 85–90 years.

In addition, we estimated the percentage of potentially avoided revisions by assuming that all patients under 75 years of age received their primary arthroplasty 5 years later while keeping the age-specific revision risks constant. This age was chosen in the context of the remaining life expectancy at older ages, where assuming a delay of 5 years for an 80-year-old seemed unrealistic, as well as the increasing operative risks at these older ages.

Finally, to quantify the impact of age at primary THA/TKA on OA revision surgery in the near future we projected our numbers (primary arthroplasties and revision risks) onto the expected Dutch population in 2025 and 2035. We calculated the expected relative increase in the Dutch population by dividing the expected Dutch population, estimated by Statistics Netherlands (CBS) in the different age categories in 2025 and 2035, by the existing population of 2010 as reported by CBS. We then multiplied the yearly number of primary arthroplasties (from the period 2007–2018) within each age category by these age-specific relative population increases to calculate the expected total number of primary arthroplasties for each separate category (stratified for age and fixation type) in 2025 and 2035. By applying the estimated revision risks to the expected number of primary arthroplasties and comparing these with the current number of revisions, the total amount of avoided revisions in 2025 and 2035 could be estimated.

### Ethics, funding, and potential conflicts of interest

The medical ethical committee of the Leiden University Medical Centre considered the study not to subject to the Medical Research Involving Human Subjects Act (WMO) (protocol number G18.037).

The study was funded by a grant from the Dutch Arthritis Foundation (ARGON, BP12-3-401). This foundation did not play a role in the study’s design, conduct or reporting.

The authors have no conflicts of interest to declare.

## Results

211,099 primary TKAs and 214,638 primary THAs were included, of which 95% and 31% were cemented. Most prostheses were placed in women (Table 1). The absolute numbers of arthroplasties, revision surgeries, and deaths per age category are included in Appendix 1.

**Table 1.** Baseline characteristics

	Total knee arthroplasty	Total hip arthroplasty
Factor	n = 211,099	n = 214,638
Age, mean (SD)	69.2 (8.6)	70.4 (8.6)
Male sex, n (%)	73,070 (35)	70,723 (33)
Fixation, n (%)		
Uncemented	10,598 (5)	148,362 (69)
Cemented	200,501 (95)	66,123 (31)

In TKAs, there was a strong age gradient; in both fixation groups the 10-year cumulative revision risk, with arthroplasties placed at a younger age, having a higher cumulative risk (Table 2). The lowest cumulative risks were found in cemented TKAs that were placed in males aged 85–90 years (cumulative revision risk 1.6%, 95% CI 0.96–2.6) (Table 2). The highest cumulative risks were found in uncemented TKAs placed in males aged 50–54 years (cumulative revision risk 13%, CI 8–21). For THA, a similar age gradient was found as for TKA, with higher 10-year cumulative risks for prostheses that were placed in younger patients, although less pronounced (Table 3). 10-year risk was higher for TKA than for THA (Tables 2 and 3).

**Table 3.** Cumulative revision percentages (CR) within 10 years for osteoarthritis patients with a primary total hip arthroplasty (THA)

THA	Male		Female	
	Cemented	Uncemented	Cemented	Uncemented
Age	CR (95% CI)	CR (95% CI)	CR (95% CI)	CR(95% CI)
50–54	– ^a^	5.2 (4.2–6.6)	5.3 (2.9–9.6)	6.0 (4.8–7.4)
55–59	4.3 (2.6–7.1)	5.5 (4.6–6.5)	7.3 (4.6–11.5)	5.1 (4.3–6.0)
60–64	3.5 (2.4–5.2)	4.6 (4.0–5.2)	4.7 (3.6–6.1)	4.2 (3.7–4.8)
65–69	5.4 (4.3–6.8)	4.4 (3.8–5.0)	3.7 (3.0–4.5)	3.6 (3.3–4.1)
70–74	4.2 (3.5–5.1)	4.3 (3.7–4.8)	3.1 (2.7–3.6)	4.2 (3.7–4.6)
75–79	4.1 (3.3–5.0)	4.1 (3.5–4.7)	3.2 (2.8–3.6)	4.2 (3.7–4.7)
80–84	2.8 (2.3–3.5)	3.5 (2.8–4.3)	2.2 (1.9–2.6)	3.3 (2.8–3.8)
85–90	1.7 (1.1–2.7)	3.2 (2.2–4.6)	2.1 (1.6–2.6)	3.5 (2.8–4.5)

**^a^** The subgroup of cemented THAs in males 50–54 years was too small to calculate valid 10-year cumulative risk percentages.

**Table 2.** Cumulative revision percentages (CR) within 10 years for osteoarthritis patients with a primary total knee arthroplasty (TKA)

TKA	Male		Female	
	Cemented	Uncemented	Cemented	Uncemented
Age	CR (95% CI)	CR (95% CI)	CR (95% CI)	CR(95% CI)
50–54	11 (9.3–13)	13 (8.0–21)	11 (10–13)	10 (7.1–15)
55–59	9.4 (8.5–11)	8.9 (6.2–13)	8.9 (8.1–9.8)	7.7 (5.6–11)
60–64	6.4 (5.7–7.1)	7.6 (5.6–11)	6.8 (6.3–7.4)	6.6 (4.9–8.7)
65–69	5.4 (4.9–6.0)	4.8 (3.1–7.5)	5.4 (5.1–5.9)	7.8 (6.1–9.9)
70–74	4.8 (4.3–5.4)	4.4 (2.8–7.0)	4.8 (4.5–5.2)	5.4 (3.9–7.3)
75–79	3.4 (3.0–3.9)	3.3 (2.0–5.6)	3.6 (3.3–3.9)	3.4 (2.4–5.0)
80–84	2.5 (2.1–3.1)	3.2 (1.6–6.4)	2.3 (2.1–2.6)	3.0 (1.9–4.7)
85–90	1.6 (1.0–2.6)	– ^a^	1.7 (1.3–2.2)	2.0 (0.9–4.9)

**^a^**The subgroup of uncemented TKAs in males 85–90 years was too small to calculate valid 10-year cumulative risk percentages.

We estimated that by delaying primary TKA and THA in OA patients for 5 years, 162 TKA revision (17%) and 23 THA revision surgeries (3%) could be avoided. Using the expected Dutch population in 2025 rather than the population from 2010, delaying primary TKA and THA in OA patients for 5 years, 203 (16%) TKA revision and 26 (3%) THA revision surgeries could be avoided. Projecting to the expected Dutch population in 2035 shows a slight decrease in these numbers because of the change in age distribution; in 2035 the Dutch population will consist of more elderly people compared with the years before (Table 4).

**Table 4.** Avoided revision surgeries in the Netherlands by delaying primary arthroplasty by 5 years

	Avoided revision surgeries, n		
Type	2010	2025	2035
Total knee arthroplasty	162	203	198
Total hip arthroplasty	23	26	20

## Discussion

In this nationwide population-based registry study we estimated the 10-year cumulative revision risks stratified by joint, age, sex, and fixation of prosthesis for OA to provide a simple tool to help to estimate the revision risk when considering arthroplasty.

The 10-year cumulative revision risks varied between 1.6% (male cemented TKA patients aged 85–90 years) and 13% (male uncemented TKA patients aged 50–54 years). The cumulative 10-year revision risks decreased by age irrespective of sex and fixation type. The age gradient was less pronounced in THA than in TKA patients. Delaying primary TKA and THA surgery by 5 years in patients under 75 years of age was estimated to avoid 3% of THA revisions and 17% of TKA revisions. Elderly patients may have a higher revision risk in the first year after arthroplasty, especially when they are frail and have various comorbidities (Johnson et al. 2019, Peters et al. 2019). However, we found that the long-term cumulative revision risks of arthroplasties placed in elderly patients were lower in all our categories than in younger patients. This finding is in accordance with previous findings (Julin et al. 2010, Wainwright et al. 2011, Carr et al. 2012, Ackerman et al. 2017a, Bayliss et al. 2017, SKAR 2017) although previous studies often did not take into account the competing risk of dying.

The choice of taking into account competing risks has been debated for several years within arthroplasty register societies and depends on the perspective taken (Van Der Pas et al. 2018). When the competing risk of dying is not taken into account this answers the question “What would happen if the competing event could be prevented [from occurring], creating an imaginary world in which an individual remains at risk of failure from the event of interest” (Putter et al. 2007), in this case the risk of revision if there is no mortality, which is appropriate when considering the perspective on which implant would have the best longevity or for etiological questions (Sayers et al. 2018, Van Der Pas et al. 2018). However, one can argue as to whether this is appropriate when communicating absolute revision risks to patients, as then the risk of death is also of interest and should be included in the estimates (Koller et al. 2012, Lacny et al. 2015, Wongworawat et al. 2015, Ranstam and Robertsson 2017, Sayers et al. 2018). When including competing risks, a different question is answered: “What is the absolute risk of revision surgery as observed in practice?” For the latter, the mortality pattern of the underlying population is also taken into account, which might be important particularly in older patients where mortality risks are higher. Because individuals who die before experiencing a revision are censored in estimates that do not take competing risks into account while they are included in competing risks analyses, revision risks are lower in practice when taking into account death as a competing risk. We have chosen to use competing risk analysis as our aim was to present the “real life” observed 10-year revision risks in practice. Here the underlying mortality patterns of the different age groups are an essential underlying process (Koller et al. 2012, Sayers et al. 2018).

For optimal timing of primary arthroplasty, it seems beneficial to postpone the time to arthroplasty to decrease the risk of revision surgery and thereby optimize the overall outcome across the entire life course, given that outcomes after revision surgery are often worse than after primary surgery. However, only revision surgery was taken into account as an outcome in this study, which implicitly also takes into account any underlying patient and surgeon preference to perform the revision, whereas other relevant outcomes such as patient-reported outcomes were not included. If delaying the primary surgery means that patients experience decreased functioning or increased pain during these years, then we may need to reconsider whether lifetime outcomes are in fact better. In the last decade, registers and cohort studies have started the assessment of patient-reported outcomes, but these are usually assessed only in the first year after primary arthroplasty and long-term outcomes as well as patient-reported outcomes after revision surgery are scarce. Such PROMs data would be valuable to compare long-terms PROMs after primary surgery with PROMs after revision surgery to inform patients on the extent of benefit they will attain, and thus be part of the decision-making on weighing risks and benefits. However, caution is required as, with time, other factors like comorbidities or ageing may also affect the PROMs and should not be attributed to surgery long ago. Moreover, we showed that delaying primary TKA and THA surgery by 5 years in the groups aged younger than 75 reduced the number of revision surgeries and thereby potentially their associated costs. However, one should bear in mind that these patients will likely need other treatment instead. For instance, patients could be offered physical therapy and additional pain medication to cope with their OA complaints. Postponing surgery may also lead to costs due to loss of productivity in patients who are still of working age.

Our study should be interpreted with its strengths and limitations in mind. One of its strengths is that our study is based on population-based data from the Dutch Arthroplasty Register, a nationwide registry that contains over 95% of primary hip and knee arthroplasties since 2010 in the Netherlands (van Steenbergen et al. 2015). The registry-based nature of the data implies that we have information only on revision surgery that was registered. Nevertheless, we consider this information bias regarding surgery was no big issue here as the completeness of TKA and THA revision arthroplasties in the LROI has been over 85% since 2012 and reached 98% in 2017 (www.lroi-report.nl).

A limitation is that we only took revision surgery into account and did not include other patient outcomes. For some patients it may not be possible to delay surgery for 5 years as functional complaints and pain may become too disabling, so that we will have overestimated the number of avoided revisions. Moreover, data are surgeon-reported revision risks that represent daily clinical practice. Hence, as indication criteria for revision surgery are not clearly defined and may vary between different surgeons, this could mean that in 2 similar patients 1 will receive a revision whereas the other will not. This is reinforced further because treatment preferences may vary between patients as well as by sex and age (Mota et al. 2012). In certain cases the physical condition of a patient will not allow revision surgery although it is indicated, which is likely to occur more often in elderly patients. As such, the patients receiving revision surgery will not include all patients in need of revision surgery. Also, we presented only the cumulative 10-year revision risks and risks over an even longer period (e.g., 20 years) may be substantially higher, especially in the younger age groups. For instance, Bayliss et al. (2017) found, in a study in which they predicted lifetime revision risks, that men who had their initial primary TKA surgery between the age of 50 and 54 years had a lifetime revision risk of 35%, considerably higher than the 10-year risks we found, but without taking competing mortality risks into account and this is therefore likely overestimated. Our nationwide population-based study has the advantage of including far more age groups as well as specific fixation groups, to make cumulative revision risks ready to be used by patients and surgeons in daily practice to improve decision-making regarding timing of primary surgery.

In conclusion, in this nationwide study we found that in both TKAs and THAs the cumulative 10-year revision risk percentages varied considerably by age, irrespective of sex and fixation of the prosthesis, but with a stronger age gradient for TKAs. By delaying the primary arthroplasty, revision procedures might be avoided, resulting in substantial revision reductions.

## Supplementary Material

Supplemental Material

## Supplementary data

Appendix is available as supplementary data in the online version of this article, http://dx.doi.org/10.1080/17453674.2021.1876998
