# Supplementary material for: Population-based 10-year cumulative revision risks after hip and knee arthroplasty for osteoarthritis to inform patients in clinical practice: a competing risk analysis from the Dutch Arthroplasty Register
Source: Acta Orthop. 2021 Jan 22;92(3):280–4. doi: 10.1080/17453674.2021.1876998 (PMC8237834; doi:10.1080/17453674.2021.1876998)
Supplement: Supplemental Material [file IORT_A_1876998_SM0561.pdf]

Supplementary data

Appendix. Absolute numbers: included arthroplasties, revision surgeries and deaths per age category

	Male						Female					
	Cemented			Uncemented			Cemented			Uncemented		
Age	Primary arthroplasties	Revisions	Deaths	Primary arthroplasties	Revisions	Deaths	Primary arthroplasties	Revisions	Deaths	Primary arthroplasties	Revisions	Deaths
TKA												
50–54	3,603	242	65	185	17	3	5,983	432	77	327	4	27
55–59	7,928	487	199	454	33	17	11,882	657	214	657	40	13
60–64	12,960	545	473	640	40	24	19,365	848	455	1,057	55	37
65–69	14,687	515	734	740	25	45	25,674	924	917	1,276	72	51
70–74	13,841	434	1,129	742	21	84	26,489	859	1,419	1,327	52	70
75–79	10,146	241	1,341	547	15	97	22,865	632	2,308	1,223	31	141
80–84	4,892	101	1,133	298	8	83	14,306	282	2,385	786	20	192
85–90	1,329	17	397	78	0	35	4,551	70	1,169	261	5	73
THA												
50–54	192	14	6	3,545	113	61	311	12	17	4,488	153	64
55–59	420	15	17	6,363	227	173	777	35	38	9,055	281	169
60–64	990	30	58	10,077	315	374	2,261	76	91	16,136	429	438
65–69	2,170	86	188	12,424	376	667	5,796	131	342	21,645	550	766
70–74	4,020	127	436	10,484	319	850	11,248	254	908	19,272	541	964
75–79	4,677	137	823	6,764	203	915	14,212	314	1,676	14,057	402	1,338
80–84	3,332	81	851	3,157	94	629	10,510	181	1,920	7,390	196	1,180
85–90	1,130	18	380	972	27	299	4,191	70	1,106	2,532	74	646
